# 5,11-Dimethyl-6,12-dimeth­oxy­indolo[3,2-*b*]carbazole

**DOI:** 10.1107/S1600536813001463

**Published:** 2013-01-19

**Authors:** Norma Wrobel, Bernhard Witulski, Dieter Schollmeyer, Heiner Detert

**Affiliations:** a University Mainz, Duesbergweg 10-14, 55099 Mainz, Germany; b Laboratoire de Chimie Moléculaire et Thio-organique, UMR 6507, ENSICAEN, 6 Boulevard Maréchal Juin, 14050 Caen, France

## Abstract

The title compound, C_22_H_20_N_2_O_2_, was prepared in a twofold Cadogan cyclization followed by double *N*-methyl­ation. The crystal structure is characterized by a zigzag arrangement of centrosymmetric mol­ecules. The indolocarbazole framework is essentially planar [maximum deviation = 0.028 (2) Å] and the meth­oxy groups are orthogonal to this plane [C—C—O—C torsion angle = −88.2 (2)°]. The lengths of the C—N bonds are nearly identical and all C—C bonds of the pyrrole subunit are significantly longer than the C—C bonds in the benzene rings.

## Related literature

For the synthesis of starting material see: Wrobel *et al.* (2012 ▶). For the Cadogan reaction, see: Cadogan (1962 ▶); Peng *et al.* (2011 ▶). For other approaches to indolocarbazoles, see: Knölker & Reddy (2002 ▶); Katritzky *et al.* (1995 ▶). For the structure of *N*-unsubstituted indolocarbazole, see: Wrobel *et al.* (2013 ▶). For electronic properties of indolocarbazoles, see: Hu *et al.* (1999 ▶); Wakim *et al.* (2004 ▶); Nemkovich *et al.* (2009 ▶). For heteroanalogous carbazoles, see: Dassonneville *et al.* (2011 ▶); Letessier & Detert (2012 ▶); Nissen & Detert (2011 ▶); Letessier *et al.* (2012 ▶). For conjugated oligomers, see: Detert *et al.* (2010 ▶).
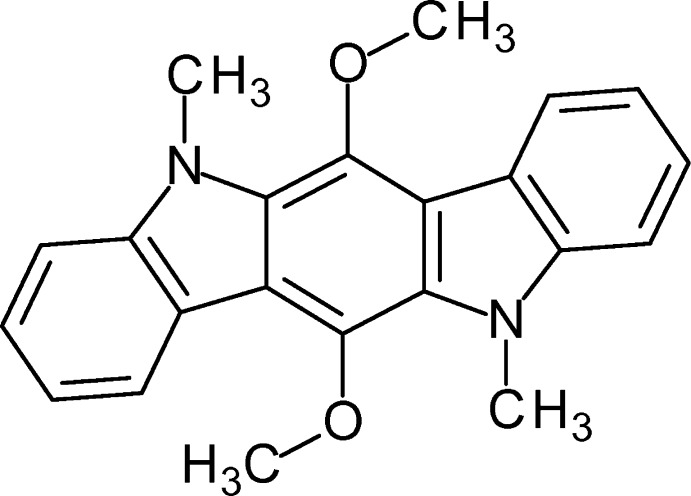


## Experimental

### 

#### Crystal data

C_22_H_20_N_2_O_2_
*M* *_r_* = 344.40
Monoclinic, 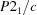

*a* = 11.229 (4) Å
*b* = 7.8561 (7) Å
*c* = 9.668 (3) Å
β = 94.790 (17)°
*V* = 849.9 (4) Å^3^
*Z* = 2
Cu *K*α radiation
μ = 0.69 mm^−1^
*T* = 193 K
0.30 × 0.30 × 0.18 mm

#### Data collection

Enraf–Nonius CAD-4 diffractometer
1716 measured reflections
1612 independent reflections
1410 reflections with *I* > 2σ(*I*)
*R*_int_ = 0.029
3 standard reflections every 60 min intensity decay: 4%

#### Refinement

*R*[*F* ^2^ > 2σ(*F* ^2^)] = 0.049
*wR*(*F* ^2^) = 0.152
*S* = 1.10
1612 reflections
120 parameters
H-atom parameters constrained
Δρ_max_ = 0.25 e Å^−3^
Δρ_min_ = −0.27 e Å^−3^

### 

Data collection: *CAD-4 Software* (Enraf–Nonius, 1989 ▶); cell refinement: *CAD-4 Software*; data reduction: *CORINC* (Dräger & Gattow, 1971 ▶); program(s) used to solve structure: *SIR97* (Altomare *et al.*, 1999 ▶); program(s) used to refine structure: *SHELXL97* (Sheldrick, 2008 ▶); molecular graphics: *PLATON* (Spek, 2009 ▶); software used to prepare material for publication: *PLATON*.

## Supplementary Material

Crystal structure: contains datablock(s) I, global. DOI: 10.1107/S1600536813001463/bt6882sup1.cif

Structure factors: contains datablock(s) I. DOI: 10.1107/S1600536813001463/bt6882Isup2.hkl

Supplementary material file. DOI: 10.1107/S1600536813001463/bt6882Isup3.cml

Additional supplementary materials:  crystallographic information; 3D view; checkCIF report

## Figures and Tables

**Fig. 1.**
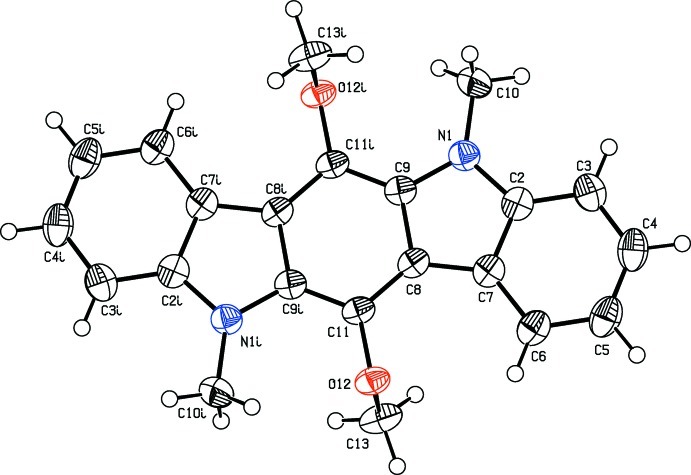
Crystal structure of the title compound with labeling and displacement ellipsoids drawn at the 50% probability level. Symmetry code: i = 1 - x, 1 - y, 1 - z.

## supplementary crystallographic information

### Comment

As part of a larger project on the synthesis of carbazoles (Letessier & Detert, 2012) and carbolines (Dassonneville *et al.* 2011; Nissen & Detert, 2011; Letessier *et al.* 2012) indolo-annulated carbazoles were prepared for optoelectronic applications. The title compound is crystallographically centrosymmetric. The pentacyclic indolocarbazole framework is essentially planar with maximum deviations of 0.028 (2) Å from the mean plane. The dihedral angle between the mean plane of the aromatic system and the adjacent *O*-methyl unit (C8—C11—O12—C13) is -88.2 (2)°. The lengths of the C—N bond are nearly identical (N1—C2: = 1.378 (2) Å, N1—C9 = 1.392 (2) Å) and all CC bonds of the pyrrole subunit (C2—C7 = 1.412 (3) Å, C7—C8 = 1.442 (3) Å, 1.423 (2) Å) are significantly longer than the CC bonds in the benzene rings (C2—C3 = 1.396 (3) Å, 1.382 (3) Å, C4—C5 = 1.395 (3) Å, C5—C6 = 1.391 (3) Å, C6—C7 = 1.397 (3) Å, C8—C11 = 1.393 (3) Å, C9—C11 = 1.391 (2) Å).

### Experimental

5,11-Dimethyl-6,12-dimethoxyindolo[3,2-*b*]carbazole was prepared from 1,4-dimethoxy-2,5-bis(2-nitrophenyl)benzene (prepared analogous to Wrobel *et al.* 2012) *via* Cadogan cyclization. In a microwave reactor tube 300 mg of the dinitro-compound were mixed with triethyl phosphite (3 ml) and irradiated (300 W, 483 K) for 15 min. The cooled mixture was dissolved in ethyl acetate (50 ml), and the same amount of hydrochloric acid (6 N) was added and the mixture heated for 3 h to reflux. After dilution with water, the product was extracted with dichloromethane (3x), the pooled organic solutions were washed with brine, dried (MgSO_4_), and concentrated. Purification by column chromatography (SiO_2_, petroleum ether/ethyl acetate = 3/1 (*v*/*v*), *R*_f_ = 0.69). Yield: 664 mg (97%) of a brownish solid with m.p. = 530–531 K. Single crystals were grown by recrystallization from dichloromethane/propanol-2.

### Refinement

Hydrogen atoms attached to carbons were placed at calculated positions with C—H = 0.95 Å (aromatic) or 0.98–0.99 Å (*sp*^3^ C-atom). All H atoms were refined in the riding-model approximation with isotropic displacement parameters set at 1.2–1.5 times of the *U*_eq_ of the parent atom.

### Crystal data

**Table:**

C_22_H_20_N_2_O_2_	*F*(000) = 364
*M**_r_* = 344.40	*D*_x_ = 1.346 Mg m^−^^3^
Monoclinic, *P*2_1_/*c*	Melting point: 530 K
Hall symbol: -P 2ybc	Cu *K*α radiation, λ = 1.54178 Å
*a* = 11.229 (4) Å	Cell parameters from 25 reflections
*b* = 7.8561 (7) Å	θ = 30–44°
*c* = 9.668 (3) Å	µ = 0.69 mm^−^^1^
β = 94.790 (17)°	*T* = 193 K
*V* = 849.9 (4) Å^3^	Plate, colourless
*Z* = 2	0.30 × 0.30 × 0.18 mm

### Data collection

**Table:**

Enraf–Nonius CAD-4 diffractometer	*R*_int_ = 0.029
Radiation source: rotating anode	θ_max_ = 70.0°, θ_min_ = 4.0°
Graphite monochromator	*h* = −13→13
ω/2θ scans	*k* = −9→0
1716 measured reflections	*l* = −11→0
1612 independent reflections	3 standard reflections every 60 min
1410 reflections with *I* > 2σ(*I*)	intensity decay: 4%

### Refinement

**Table:**

Refinement on *F*^2^	Primary atom site location: structure-invariant direct methods
Least-squares matrix: full	Secondary atom site location: difference Fourier map
*R*[*F*^2^ > 2σ(*F*^2^)] = 0.049	Hydrogen site location: inferred from neighbouring sites
*wR*(*F*^2^) = 0.152	H-atom parameters constrained
*S* = 1.10	*w* = 1/[σ^2^(*F*_o_^2^) + (0.0969*P*)^2^ + 0.1743*P*] where *P* = (*F*_o_^2^ + 2*F*_c_^2^)/3
1612 reflections	(Δ/σ)_max_ < 0.001
120 parameters	Δρ_max_ = 0.25 e Å^−^^3^
0 restraints	Δρ_min_ = −0.27 e Å^−^^3^

### Special details

**Table:**

Experimental. H-NMR (400 MHz, CDCl_3_): 8.25 (d, J = 7.7 Hz, 2 H), 7.58 (d, J = 7.7 Hz, 2 H), 7.50 (dt, J = 7.7 Hz, J= 1.0 Hz, 2 H), 7.26 - 7.22 (m, 2 H), 4.17 (s, 6 H, CH_3_), 4.15 (s, CH_3_).C-NMR (75 MHz, CDCl_3_): 145.2 (*s*), 136.4 (*s*), 128.5 (*s*), 125.6 (*d*), 122.7 (*d*), 121.5 (*s*), 118.7 (*d*), 117.8 (*s*), 108.0 (*d*), 61.8 (*q*), 31.2 (*q*).IR (ATR) 3043, 2926, 2850, 2828, 1733, 1608, 1530, 1465, 1438, 1390, 1324, 1289, 1247, 1200, 1154, 1117, 1078, 1006, 933 cm^-1^.MS (EI): 344 (100%) [*M*]^+^.ESI-HRMS: C_22_H_21_N_2_O_2_ calcd.: 345.1603, found 345.1580.UV-Vis (dichloromethane): λ = 393 nm, λ_max_ = 412 nm; Fluorescence: λ_max_ = 428 nm (dichloromethane).
Geometry. All e.s.d.'s (except the e.s.d. in the dihedral angle between two l.s. planes) are estimated using the full covariance matrix. The cell e.s.d.'s are taken into account individually in the estimation of e.s.d.'s in distances, angles and torsion angles; correlations between e.s.d.'s in cell parameters are only used when they are defined by crystal symmetry. An approximate (isotropic) treatment of cell e.s.d.'s is used for estimating e.s.d.'s involving l.s. planes.
Refinement. Refinement of *F*^2^ against ALL reflections. The weighted *R*-factor *wR* and goodness of fit *S* are based on *F*^2^, conventional *R*-factors *R* are based on *F*, with *F* set to zero for negative *F*^2^. The threshold expression of *F*^2^ > σ(*F*^2^) is used only for calculating *R*-factors(gt) *etc*. and is not relevant to the choice of reflections for refinement. *R*-factors based on *F*^2^ are statistically about twice as large as those based on *F*, and *R*- factors based on ALL data will be even larger.

### Fractional atomic coordinates and isotropic or equivalent isotropic displacement parameters (Å^2^)

**Table:**

	*x*	*y*	*z*	*U*_iso_*/*U*_eq_	
N1	0.25894 (13)	0.42605 (18)	0.48867 (15)	0.0346 (4)	
C2	0.21698 (16)	0.4933 (2)	0.36211 (18)	0.0342 (4)	
C3	0.10013 (17)	0.4953 (2)	0.3004 (2)	0.0416 (5)	
H3	0.0365	0.4447	0.3445	0.050*	
C4	0.08055 (19)	0.5736 (3)	0.1724 (2)	0.0476 (5)	
H4	0.0018	0.5763	0.1282	0.057*	
C5	0.1729 (2)	0.6487 (2)	0.1064 (2)	0.0475 (5)	
H5	0.1561	0.7023	0.0188	0.057*	
C6	0.28932 (18)	0.6459 (2)	0.16765 (19)	0.0397 (5)	
H6	0.3524	0.6961	0.1223	0.048*	
C7	0.31206 (16)	0.56790 (19)	0.29716 (18)	0.0330 (4)	
C8	0.41824 (15)	0.54265 (19)	0.38964 (17)	0.0303 (4)	
C9	0.38140 (15)	0.45609 (19)	0.50808 (17)	0.0307 (4)	
C10	0.18776 (17)	0.3313 (3)	0.5793 (2)	0.0480 (5)	
H10A	0.1158	0.2886	0.5262	0.072*	
H10B	0.1648	0.4056	0.6540	0.072*	
H10C	0.2344	0.2351	0.6194	0.072*	
C11	0.53766 (15)	0.58538 (19)	0.38040 (17)	0.0306 (4)	
O12	0.57194 (11)	0.66686 (14)	0.26343 (12)	0.0364 (4)	
C13	0.6004 (2)	0.5500 (3)	0.15737 (19)	0.0480 (5)	
H13A	0.6734	0.4877	0.1882	0.072*	
H13B	0.6128	0.6130	0.0723	0.072*	
H13C	0.5344	0.4693	0.1391	0.072*	

### Atomic displacement parameters (Å^2^)

**Table:**

	*U*^11^	*U*^22^	*U*^33^	*U*^12^	*U*^13^	*U*^23^
N1	0.0362 (8)	0.0301 (7)	0.0376 (8)	−0.0027 (6)	0.0035 (6)	0.0014 (6)
C2	0.0405 (9)	0.0242 (8)	0.0376 (9)	0.0030 (7)	0.0014 (7)	−0.0048 (7)
C3	0.0375 (9)	0.0346 (9)	0.0524 (11)	0.0047 (7)	0.0018 (8)	−0.0054 (8)
C4	0.0440 (10)	0.0399 (10)	0.0564 (12)	0.0108 (8)	−0.0101 (9)	−0.0038 (9)
C5	0.0569 (12)	0.0346 (10)	0.0486 (11)	0.0073 (8)	−0.0107 (9)	0.0039 (8)
C6	0.0507 (11)	0.0269 (8)	0.0404 (10)	0.0003 (7)	−0.0032 (8)	0.0018 (7)
C7	0.0414 (9)	0.0209 (7)	0.0362 (9)	−0.0002 (6)	0.0008 (7)	−0.0032 (6)
C8	0.0414 (9)	0.0197 (7)	0.0297 (8)	−0.0015 (6)	0.0018 (6)	−0.0019 (6)
C9	0.0381 (9)	0.0211 (7)	0.0332 (9)	−0.0027 (6)	0.0044 (7)	−0.0023 (6)
C10	0.0382 (10)	0.0556 (12)	0.0509 (12)	−0.0064 (9)	0.0085 (8)	0.0114 (9)
C11	0.0425 (9)	0.0202 (7)	0.0295 (8)	−0.0030 (6)	0.0049 (7)	0.0001 (6)
O12	0.0488 (8)	0.0283 (6)	0.0327 (7)	−0.0052 (5)	0.0060 (5)	0.0053 (5)
C13	0.0740 (14)	0.0391 (10)	0.0323 (10)	−0.0070 (9)	0.0123 (9)	0.0010 (7)

### Geometric parameters (Å, º)

**Table:**

N1—C2	1.379 (2)	C7—C8	1.443 (2)
N1—C9	1.392 (2)	C8—C11	1.392 (2)
N1—C10	1.442 (2)	C8—C9	1.423 (2)
C2—C3	1.396 (3)	C9—C11^i^	1.390 (2)
C2—C7	1.410 (3)	C10—H10A	0.9800
C3—C4	1.383 (3)	C10—H10B	0.9800
C3—H3	0.9500	C10—H10C	0.9800
C4—C5	1.393 (3)	C11—O12	1.3818 (19)
C4—H4	0.9500	C11—C9^i^	1.390 (2)
C5—C6	1.390 (3)	O12—C13	1.432 (2)
C5—H5	0.9500	C13—H13A	0.9800
C6—C7	1.398 (2)	C13—H13B	0.9800
C6—H6	0.9500	C13—H13C	0.9800
			
C2—N1—C9	108.36 (14)	C11—C8—C7	132.65 (16)
C2—N1—C10	124.86 (16)	C9—C8—C7	106.48 (15)
C9—N1—C10	126.66 (15)	C11^i^—C9—N1	129.75 (16)
N1—C2—C3	128.54 (17)	C11^i^—C9—C8	121.42 (16)
N1—C2—C7	109.81 (16)	N1—C9—C8	108.83 (15)
C3—C2—C7	121.65 (17)	N1—C10—H10A	109.5
C4—C3—C2	117.46 (19)	N1—C10—H10B	109.5
C4—C3—H3	121.3	H10A—C10—H10B	109.5
C2—C3—H3	121.3	N1—C10—H10C	109.5
C3—C4—C5	121.99 (19)	H10A—C10—H10C	109.5
C3—C4—H4	119.0	H10B—C10—H10C	109.5
C5—C4—H4	119.0	O12—C11—C9^i^	122.33 (15)
C6—C5—C4	120.49 (19)	O12—C11—C8	119.98 (15)
C6—C5—H5	119.8	C9^i^—C11—C8	117.69 (16)
C4—C5—H5	119.8	C11—O12—C13	112.52 (13)
C5—C6—C7	118.92 (19)	O12—C13—H13A	109.5
C5—C6—H6	120.5	O12—C13—H13B	109.5
C7—C6—H6	120.5	H13A—C13—H13B	109.5
C6—C7—C2	119.49 (17)	O12—C13—H13C	109.5
C6—C7—C8	134.00 (17)	H13A—C13—H13C	109.5
C2—C7—C8	106.50 (15)	H13B—C13—H13C	109.5
C11—C8—C9	120.87 (15)		
			
C9—N1—C2—C3	−179.06 (16)	C6—C7—C8—C9	178.78 (17)
C10—N1—C2—C3	4.7 (3)	C2—C7—C8—C9	−1.05 (18)
C9—N1—C2—C7	0.16 (18)	C2—N1—C9—C11^i^	179.07 (16)
C10—N1—C2—C7	−176.07 (16)	C10—N1—C9—C11^i^	−4.8 (3)
N1—C2—C3—C4	179.03 (17)	C2—N1—C9—C8	−0.84 (18)
C7—C2—C3—C4	−0.1 (3)	C10—N1—C9—C8	175.30 (16)
C2—C3—C4—C5	−0.1 (3)	C11—C8—C9—C11^i^	1.4 (3)
C3—C4—C5—C6	0.5 (3)	C7—C8—C9—C11^i^	−178.75 (14)
C4—C5—C6—C7	−0.6 (3)	C11—C8—C9—N1	−178.63 (14)
C5—C6—C7—C2	0.4 (2)	C7—C8—C9—N1	1.17 (18)
C5—C6—C7—C8	−179.45 (18)	C9—C8—C11—O12	178.89 (13)
N1—C2—C7—C6	−179.29 (15)	C7—C8—C11—O12	−0.9 (3)
C3—C2—C7—C6	−0.0 (2)	C9—C8—C11—C9^i^	−1.4 (3)
N1—C2—C7—C8	0.57 (18)	C7—C8—C11—C9^i^	178.86 (16)
C3—C2—C7—C8	179.85 (15)	C9^i^—C11—O12—C13	92.20 (19)
C6—C7—C8—C11	−1.5 (3)	C8—C11—O12—C13	−88.10 (19)
C2—C7—C8—C11	178.72 (17)		

Symmetry code: (i) −*x*+1, −*y*+1, −*z*+1.

## References

[bb1] Altomare, A., Burla, M. C., Camalli, M., Cascarano, G. L., Giacovazzo, C., Guagliardi, A., Moliterni, A. G. G., Polidori, G. & Spagna, R. (1999). *J. Appl. Cryst.* **32**, 115–119.

[bb2] Cadogan, J. I. G. (1962). *Q. Rev.* **16**, 208–239.

[bb3] Dassonneville, B., Witulski, B. & Detert, H. (2011). *Eur. J. Org. Chem.* pp. 2836–2844.

[bb4] Detert, H., Lehmann, M. & Meier, H. (2010). Materials, **3**, 3218–3330.

[bb5] Dräger, M. & Gattow, G. (1971). *Acta Chem. Scand.* **25**, 761–762.

[bb6] Enraf–Nonius (1989). *CAD-4 Software* Enraf–Nonius, Delft, The Netherlands.

[bb7] Hu, N.-X., Xie, S., Popovic, Z., Ong, B. & Hor, A.-M. (1999). *J. Am. Chem. Soc.* **121**, 5097–5098.

[bb8] Katritzky, A. R., Li, J. & Stevens, C. V. (1995). *J. Org. Chem.* **60**, 3401–3404.

[bb9] Knölker, H.-J. & Reddy, K. R. (2002). *Chem. Rev.* **39**, 6521–6527.

[bb10] Letessier, J. & Detert, H. (2012). *Synthesis*, **44**, 290–296.

[bb11] Letessier, J., Detert, H., Götz, K. & Opatz, T. (2012). *Synthesis*, **44**, 747–754.

[bb12] Nemkovich, N. A., Kruchenok, Yu. V., Sobchuk, A. N., Detert, H., Wrobel, N. & Chernyavskii, E. A. (2009). *Opt. Spectrosc.* **107**, 275–281.

[bb13] Nissen, F. & Detert, H. (2011). *Eur. J. Org. Chem.* pp. 2845–2854.

[bb14] Peng, H., Chen, X., Chen, Y., He, Q., Xie, Y. & Yang, C. (2011). *Tetrahedron*, **67**, 5725–5731.

[bb15] Sheldrick, G. M. (2008). *Acta Cryst.* A**64**, 112–122. 10.1107/S0108767307043930 18156677

[bb16] Spek, A. L. (2009). *Acta Cryst.* D**65**, 148–155. 10.1107/S090744490804362X PMC2631630 19171970

[bb17] Wakim, S., Bouchard, J., Simard, M., Drolet, N., Tao, Y. & Leclerc, M. (2004). *Chem. Mater.* **16**, 4386–4388.

[bb18] Wrobel, N., Schollmeyer, D. & Detert, H. (2012). *Acta Cryst.* E**68**, o1022. 10.1107/S1600536812009944 PMC3343986 22589895

[bb19] Wrobel, N., Witulski, B., Schollmeyer, D. & Detert, H. (2013). *Acta Cryst.* E**69**, o116–o117. 10.1107/S1600536812050611 PMC3588340 23476379

